# Efficient Multi-Enzymes Immobilized on Porous Microspheres for Producing Inositol From Starch

**DOI:** 10.3389/fbioe.2020.00380

**Published:** 2020-05-05

**Authors:** Pingping Han, Xigui Zhou, Chun You

**Affiliations:** ^1^Tianjin Institute of Industrial Biotechnology, Chinese Academy of Sciences, Tianjin, China; ^2^University of Chinese Academy of Sciences, Beijing, China

**Keywords:** multi-enzymes, immobilization, inositol, porous microspheres, cascade biocatalysis

## Abstract

*In vitro* synthetic enzymatic biosystem is considered to be the next generation of biomanufacturing platform. This biosystem contains multiple enzymes for the implementation of complicated biotransformatiom. However, the hard-to-reuse and instability of multiple enzymes limit the utilization of this biosystem in industrial process. Multi-enzyme immobilization might be a feasible alternative to address these problems. Herein, porous microspheres are used as carriers to co-immobilize multiple enzymes for producing inositol from starch. At first, all the enzymes (i.e., α-glucan phosphorylase aGP, phosphoglucose mutase PGM, inositol 1-phosphate synthase IPS, and inositol monophosphatase IMP) for converting starch to inositol were immobilized on porous microspheres individually to check the effect of immobilization, then all the enzymes are co-immobilized on porous microspheres. Compared to reaction system containing all the individual immobilized enzymes, the reaction system containing the co-immobilized enzymes exhibit ∼3.5 fold of reaction rate on producing inositol from starch. This reaction rate is comparable to that by free enzyme mixture. And the co-immobilized multi-enzyme system show higher thermal stability and recovery stability than free enzyme mixture. After 7 batches, the immobilized enzymes retain 45.6% relative yield, while the free enzyme mixture only retain 13.3% relative yield after 3 batches. Co-immobilization of multiple enzymes on porous microspheres for biomanufacturing would shed light on the application of *in vitro* synthetic enzymatic biosystem in industrial scale.

## Introduction

**FIGURE 1:**
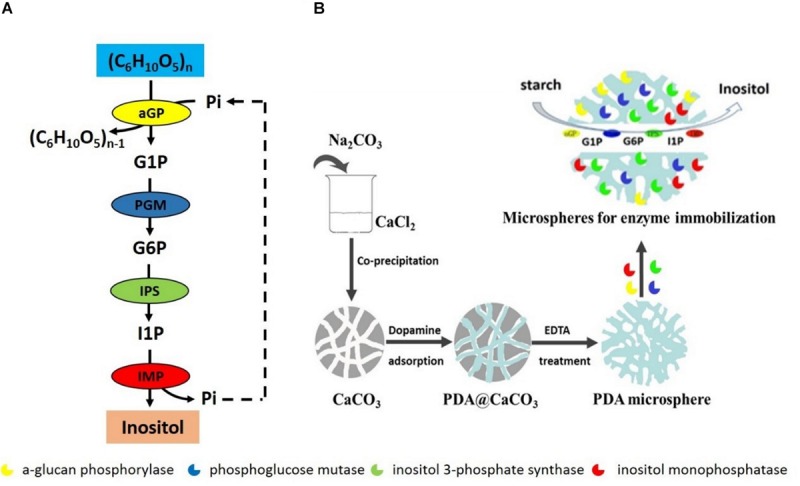
**(A)** Scheme of *in vitro* synthetic enzymatic pathway for inositol synthesis from starch. **(B)** The scheme preparation process of immobilized enzymes on porous microspheres.

*In vitro* synthetic enzymatic biosystem refers to the use of several enzymes and co-enzymes in a cascade for the production of desired compounds (Paloma et al., 2011; Zhang Y.P. et al., 2017). This biosystem represents the next generation of biomanufacturing for many advantages compared to the microbial fermentation, which is current dominant biomanufacturing platform. These advantages include high product yield, high reaction rate, highly engineering flexibility, and easy industrial scale-up and better system robustness (You et al., 2013; Zhang, 2015; Zhang Y.P. et al., 2017). Many *in vitro* synthetic enzymatic biosystems have been designed and constructed to produce various chemicals from low to high value, such as hydrogen, inositol, antibiotics, and antitumor drugs (Kim et al., 2017, 2018; Lee et al., 2017; Wang et al., 2017; You et al., 2017; Lu et al., 2018). Recently, an *in vitro* synthetic enzymatic biosystem of producing inositol has been successfully scaled up in a reactor of 20 ton (You et al., 2017). This enzymatic biosystem contains four key enzymes: α-glucan phosphorylase (αGP, EC 2.4.1.1), phosphoglucose mutase (PGM, EC 5.4.2.2), inositol 1-phosphate synthase (IPS, EC 5.5.1.4), and inositol monophosphatase (IMP, EC 3.1.3.1), converting starch into inositol (Figure 1A). αGP catalyzes starch to glucose 1-phosphate (G1P) in the presence of inorganic phosphate, PGM catalyzes G1P to glucose 6-phosphate (G6P), IPS converts G6P to inositol 1-phosphate (I1P), and inositol is produced by dephosphorization of I1P catalyzed by IMP, releasing inorganic phosphate, which can be reused by αGP to produce G1P. As the last two steps catalyzed by IPS and IMP are irreversible, resulting in high inositol product yield from starch.

For applying *in vitro* synthetic enzymatic biosystem in industrial scale, enzyme cost accounts for one of the most important parts. The enzyme cost was determined by many aspects, including enzyme expression level, enzyme activity, enzyme stability, and enzyme recycling. Enzyme expression level can be improved by selection of the promoters and transcriptional activation regulation as well as codon-optimization (Mechold et al., 2005; Hu et al., 2013). Enzyme activity and stability can be improved by many protein engineering methods, such as random mutagenesis, and site-saturated mutagenesis (Rha et al., 2009; Bommarius and Paye, 2013; Li G. et al., 2016; Zhou et al., 2018; Huang R. et al., 2018). Actually, enzyme recycling could be the determinant characters for enzyme cost in industrial application. Enzyme recycling can be performed by ultrafiltration using different types of membranes (Pizzichini et al., 1991; Sen et al., 2012). However, fultrafiltration increase the complexity of industrial process. Enzyme immobilization allows for easy enzyme recycling, whereas at the same time, enzyme immobilization could improve enzyme stability, and reactivity (van de Velde et al., 2000; Jiang et al., 2009; Schoffelen and van Hest, 2012; Jia et al., 2013; Sheldon and van Pelt, 2013; Liang et al., 2016; Pitzalis et al., 2017; Talekar et al., 2017; Yu et al., 2017; Zhao et al., 2018).

Many kinds of carriers have been used for enzyme immobilization, such as microspheres, nanoparticles (Ren et al., 2011; Li et al., 2014; Li Z. et al., 2016), capsules (Shi et al., 2013; Zhang S. et al., 2017), gels (Yang et al., 2017), metal-organic frameworks (MOFs; Liang et al., 2020), and magnetic nanofiber composite (Huang W.-C. et al., 2018) etc. Among them, porous microspheres have many advantages as carriers for enzyme immobilization (Cai et al., 2013). First, highly developed porous structure of the microspheres increases the specific surface area of the microspheres, allowing the microspheres to have a high enzyme loading. (Han et al., 2015; Wang et al., 2015; Han et al., 2016; Yu et al., 2017) Second, the well-developed pore structure in the microspheres can effectively promote the rapid transfer of substrate and products, reducing mass transfer inhibition, and enhancing the catalytic activity of enzymes (Liang et al., 2016). Finally, the unique pore environment within the microspheres endows the enzyme a favorable microenvironment, thereby improving the enzyme’s stability. Thus many researchers used porous microspheres for enzyme immobilization, and these advantages of porous microspheres are well suited for simultaneous immobilization of multiple enzymes (Li et al., 2010; Chao et al., 2014; Han et al., 2015; Wang et al., 2015; Han et al., 2016).

In this work, porous microspheres were used as immobilization carriers to co-immobilize all the enzymes of an *in vitro* synthetic enzymatic biosystem for inositol production from starch. In order to verify the effect of the porous microspheres as an enzyme carrier on inositol production, all the enzymes were immobilized on porous microspheres individually to check the effect of immobilization. After checking that immobilized enzymes had much higher stability than free enzymes without losing activity significantly, especially IPS that was the rate-limited enzyme. Then all the enzymes were co-immobilized on porous microspheres. The inositol production rate of co-immobilized multi-enzymes was much higher than that of the mixture of individual immobilized enzymes, this production rate of co-immobilized multi-enzymes was comparable to that of the free enzyme mixture. However, the recycle stability of co-immobilized multi-enzymes was much higher than the free enzyme mixture, showing great potential in industrial application. This study established the groundwork for the use of porous microspheres to co-immobilize cascade enzymes in *in vitro* synthetic enzymatic biosystems.

## Experimental

### Materials

All chemicals were reagent grade or higher, purchased from Sigma-Aldrich unless otherwise noted. Maltodextrin with a dextrose equivalent (DE) of about 10 was purchased from Zhucheng Dongxiao Biotechnol Co (China). The enzymes included alpha-glucan phosphorylase (αGP, EC 2.4.1.1) from *Thermotoga maritima*, (PGM, EC 5.4.2.2) from *Thermococcus kodakarensis*, (IPS EC 5.5.1.4) from *Archaeoglobus fulgidus*, and (IMP EC 3.1.3.1) from *T. maritima*. These enzymes were produced by *Escherichia coli* BL21(DE3) in our lab by T&J-A type 5L^∗^2 Parallel Bioreactor (T&J Bio-engineering Co.,LTD, Shanghai, China) as previously described (You et al., 2017).

### Enzyme Immobilization on Porous Microspheres

A typical preparation process of porous microspheres was according to the procedure reported in the previous literature (Han et al., 2015). Briefly, porous CaCO_3_ templates were firstly prepared by the co-precipitation of equivalent CaCl_2_ and Na_2_CO_3_ solution. 10 mL of 330 mM Na_2_CO_3_ solution was rapidly added into 10 mL of 330 mM CaCl_2_ solution. The mixture was stirred for 30 s. And the porous microspheres templates were then collected by centrifugation and washed with Tris-HCl solution (pH 8.5) for several times. Next, the CaCO_3_ templates were dispersed in 6 mg/mL dopamine solution under mechanical stirring for 5 h. The polydopamine loaded CaCO_3_ microspheres were finally collected and denucleated by 50 mM EDTA treatment. Thus the porous microspheres were obtained. For the immobilization of the single enzyme, a certain amount of porous microspheres (100 mg dry weight) were dispersed into the enzyme solution. The mixture of porous microspheres and enzyme was gently stirred for 2 h. Then, the enzyme-conjugated porous microspheres were collected by centrifugation (Figure 1B). For the immobilization of multi-enzymes, the four enzymes were mixed according to the protein concentration ratio of αGP: PGM: IPS: IMP = 0.5: 0.5: 3: 0.4 and then mixed with a certain amount of porous microspheres (100 mg dry weight), followed by the immobilization of a single enzyme.

### The Calculation of Enzyme Loading on Porous Microspheres

The loading amount of enzymes onto the porous microspheres was estimated according to Bradford’s method. Briefly, the absorbance at 595 nm was measured after blending the Coomassie brilliant blue reagent with free enzymes or supernatant solution from separation and washing solution. The loading capability was calculated using Eq. (1):

(1) $$\text{Loading}\,\text{capacity}=\left({\text{C}}_{1}\,{\text{V}}_{1}-{\text{C}}_{2}\,{\text{V}}_{2}\right)/\text{M}$$

where C_1_ and V_1_ represented the initial concentration and the volume of the enzymes, respectively; C_2_ and V_2_ represented the residual concentration and the volume of the enzymes in the supernatant solution, respectively; M was the total quantity of porous microspheres.

### Activity Assay of Free Enzymes and Immobilized Enzymes

The activity of free and immobilized alpha-glucan phosphorylase (αGP) was assayed at 70°C in 100 mM HEPES buffer (pH 7.2) containing 5 g/L maltodextrin and 10 mM phosphate (pH 7.2). The product glucose 1-phosphate (G1P) in the supernatant was measured by a glucose hexokinase/glucose 6-phosphate (G6P) dehydrogenase assay kit supplemented with excess PGM.

The activity of free and immobililzed PGM was assayed at 70°C in 100 mM HEPES (pH 7.2) containing 10 mM G1P. The product G6P in the supernatant was measured by a glucose hexokinase/G6P dehydrogenase assay kit.

The activity of free and immobilized IPS was assayed at 70°C in 100 mM HEPES buffer (pH 7.2) containing 10 mM G6P in the present of excess IMP. The inorganic phosphate released from I1P was measured by the mild pH phosphate assay (Saheki et al., 1985).

The activity of free and immobilized IMP was assayed 70°C in 100 mM HEPES buffer (pH 7.2) containing 10 mM G6P in the present of excess IPS. The inorganic phosphate released from I1P was measured by the mild pH phosphate assay (Saheki et al., 1985).

One unit of enzyme activity was defined as the amount of enzyme that generated 1 μmole of product per min.

The performance of free enzyme mixture, co-immobilized multi-enzyme system, and mixture of solely immobilized enzymes were characterized by one-pot biosynthesis of inositol from maltodextrin. Maltodextrin was first pretreated by isoamylase (IA) to debranch a-1,6-glucosidic linkages at 85°C for 3 h in 5 mM sodium acetate buffer (pH 5.5) containing 0.5 mM MgCl_2_. The ratio of maltodextrin and IA was 2000:1 (weight/weight). Then the inositol production process was conducted in 1% IA treated maltodextrin containing 100 mM HEPES (pH 7.2), 50 mM phosphate, 5 mM MgCl_2_ as well as free enzyme mixture, and co-immobilized multi-enzyme system and mixture of solely immobilized enzymes at 70°C. In order to compare the catalytic performance of free enzyme mixture, co-immobilized multi-enzyme system and mixture of solely immobilized enzymes, the protein content of each enzyme in the three system was αGP 0.5 mg/mL, PGM 0.5 mg/mL, IPS 3 mg/mL, and IMP 0.4 mg/mL. The protein concentration of the enzymes was the same in the three systems The concentration of inositol was determined by HPLC equipped with Bio-Rad HPX-87H column with 5 mM H_2_SO_4_ as a mobile phase and a refractive index detector.

### Thermal Stability of Free and Immobilized Enzymes

The thermal stability was evaluated by the residual activity, which was measured in the reaction buffer mentioned in the last paragraph of section “Activity assay of free enzymes and immobilized enzymes**”**, after incubating free and immobilized enzymes in 70°C or 80°C for different times. The thermal denaturation kinetics of enzymes was expressed by the first-order exponential equation, and the thermal denaturation constants (*k*_*d*_) were calculated according to Eq. (2).

(2) $$\text{A}={\text{A}}_{0}\,\text{exp}\,\left(-k_{\text{d}}\,\text{t}\right)$$

Where A was the activity of enzymes after incubation, A_0_ was the activity of enzymes before incubation and t was the incubation time.

The half-life (t_1__/__2_) value for enzyme thermal denaturation was calculated by Eq. (3).

(3) t1/2=ln⁢ 2/kd

### Recycling Stability of Free and Immobilized Enzymes

The recycling stability of immobilized enzymes was investigated by measuring the relative activity of enzymes during the process of circular reaction under the assay conditions. For the recycling of immobilized IPS, immobilized IPS was used to produce inositol from 1% IA treated maltodextrin according to the above experiment in section “Activity assay of free enzymes and immobilized enzymes**”**, and the amount of enzymes was αGP 0.5 mg/mL, PGM 0.5 mg/mL, IPS 3 mg/mL, and IMP 0.4 mg/mL. The reaction time was set at 2 h. The amount of inositol produced in the first cycle was set as 100%. After each cycle of inositol production was finished, the porous microspheres were recycled by centrifugation at 5000 *g* for 5 min. The recycled porous microspheres were washed by 100 mM HEPES (pH 7.2) containing 50 mM phosphate and 5 mM MgCl_2_ for three times, and then was further used in the next cycle of the production of inositol from new 1% IA-treated maltodextrin with the addition of new αGP 0.5 mg/mL, PGM 0.5 mg/mL, and IMP 0.4 mg/mL.

For the recycling of free enzyme mixtures, free enzyme mixtures were used to produce inositol from 1% IA treated maltodextrin according to the above experiment in section “Activity assay of free enzymes and immobilized enzymes**”**, and the amount of enzymes was αGP 0.5 mg/mL, PGM 0.5 mg/mL, IPS 3 mg/mL, and IMP 0.4 mg/mL. The reaction time was set at 2 h. The amount of inositol produced in the first cycle was set as 100%. After each cycle of inositol production was finished, the free enzyme mixtures was recovered by ultrafiltration using 10 KDa ultrafiltration tube at 4°C. The recovered free enzyme mixtures were washed by 100 mM HEPES (pH 7.2) containing 50 mM phosphate and 5 mM MgCl_2_ for three times, and then were further used in the next cycle of the production of inositol from new 1% IA-treated maltodextrin.

For the recycling of co-immobilized multi-enzymes, porous microspheres co-immobilized multi-enzymes was used to produce inositol from 1% IA treated maltodextrin according to the above experiment in section “Activity assay of free enzymes and immobilized enzymes**”**, and the amount of enzymes was αGP 0.5 mg/mL, PGM 0.5 mg/mL, IPS 3 mg/mL, and IMP 0.4 mg/mL. The reaction time was set at 2 h. The amount of inositol produced in the first cycle was set as 100%. After each cycle of inositol production was finished, the porous microspheres were recycled by centrifugation at 5000 *g* for 5 min. The recycled porous microspheres were washed by 100 mM HEPES (pH 7.2) containing 50 mM phosphate and 5 mM MgCl_2_ for three times, and then was further used to produce inositol from new 1% IA treated maltodextrin for the next cycle.

### pH Stability of Free and Immobilized Enzymes

The pH stability of free enzyme mixture and co-immobilized multi-enzymes was investigated by measuring the relative activity of enzymes after incubating in diffferent pH values (pH 5–9) for 2 h. For free enzyme mixture, free enzyme mixture was recovered by ultrafiltration after incubating in different pH conditions. For co-immobilized multi-enzymes, co-immobilized multi-enzymes was recovered by centrifugation after incubating. Then, free enzyme mixture and co-immobilized multi-enzymes were used to produce inositol from 1% IA treated maltodextrin according to the above experiment in section “Activity assay of free enzymes and immobilized enzymes**”**.

## Results and Discussion

### Analysis of *in vitro* Synthetic Enzymatic Biosystem for Inositol Production

In our research group, an *in vitro* synthetic enzymatic biosystem containing multi-enzymes was constructed before to convert starch into high-value inositol (You et al., 2017). This synthetic pathway was composed of four key enzymes:αGP, PGM, IPS, and IMP (Figure 1A). As the last two steps catalyzed by IPS and IMP were irreversible, inositol production yield of more than 90% from 10 g/L IA treated maltodextrin was achieved in our previous research (You et al., 2017). Because of the high potential of this inositol production method in industrial scale to replace the traditional method which was acid hydrolysis of phytate to inositol, co-immobilization of all the cascade enzymes might further decrease the product cost of enzymatic production of inositol.

### Immobilization of Rate-Limited Enzyme (IPS) of the Pathway for Inositol Production

In this *in vitro* biosystem for inositol production, αGP, PGM, and IMP all had at least a magnitude of higher catalytic activity than IPS, thus IPS was the rate-limiting enzyme. Before all the enzymes for inositol production were immobilized by using porous microspheres as described above (Figure 1B), the rate-limited enzyme IPS should be a starting point. If the rate-limiting enzyme could be immobilized successfully, there was a high probability of the success for the co-immobilization of all the cascade enzymes.

**FIGURE 2:**
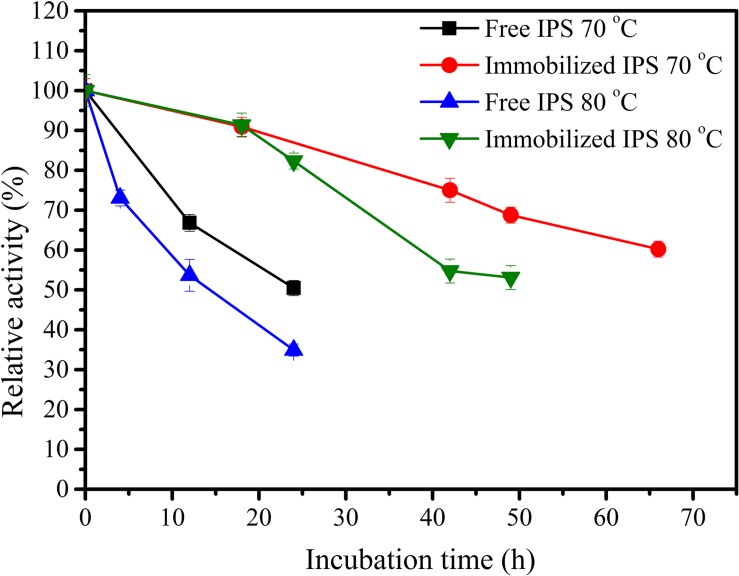
Themostability of free and immobilized IPS at 70°C and 80°C.

Inositol 1-phosphate synthase was first immobilized on porous microspheres. The loading capacity of porous microspheres to IPS could reach 266 mg protein/g carriers. Then the enzymatic characterization of immobilized IPS was carried out. As shown in Table 1, the specific activity of immobilized IPS showed a certain degree of decrease. The specific activity value of immobilized IPS was only 34.3% of the free enzyme counterpart. However, the thermal stabilities of immobilized IPS under 70°C or 80°C was much higher than free IPS. As shown in Figure 2, the relative activity of immobilized IPS was largely maintained under 70°C or 80°C. Specifically, the immobilized IPS maintained 68.8 and 82.3% residual activity after incubation at 70°C for 48 h and at 80°C for 24 h, respectively, while the free enzyme maintained only 29.5 and 34.9% residual activity, respectively. The half-lives of immobilized IPS were 3.6 and 2.7 times higher than free IPS at 70°C and 80°C, respectively (Table 2).

**TABLE 1:** Relative activity of each enzyme before and after immobilization.

	**Sp. for free enzyme U/mg**	**Sp. for immobilized enzyme U/mg**	**Residual activity%**
IPS	2.07 ± 0.1	0.71 ± 0.04	34.30 ± 1.9
αGP	16.13 ± 0.8	7.68 ± 0.3	47.57 ± 2.1
PGM	17.12 ± 0.9	10.35 ± 0.4	60.50 ± 2.6
IMP	38.47 ± 1.5	15.70 ± 0.7	40.82 ± 1.8

*Sp. represent specific activity.*

**TABLE 2:** t_1/2_ before and after immobilization at 70°C or 80°C.

	**Free enzyme (*h*) (70°C)**	**Immobilized enzyme (*h*) (70°C)**	**Free enzyme (*h*) (80°C)**	**Immobilized enzyme (*h*) (80°C)**
IPS	24.3 ± 1.4	87.7 ± 4.3	16.5 ± 0.6	47.8 ± 2.1
αGP	25.4 ± 1.2	70.0 ± 3.6	7.7 ± 0.4	34.1 ± 1.8
PGM	8.9 ± 0.8	16.9 ± 1.5	1.4 ± 0.2	3.5 ± 0.3
IMP	29.5 ± 1.8	41 ± 2.2	6.1 ± 0.3	11.0 ± 0.4

Although the specific activity of the immobilized enzyme decreased, the thermal stability of the immobilized enzyme was remarkably improved. This might be ascribed to the following reasons: The conformation of the enzyme molecule immobilized on the porous microspheres was somewhat changed compared to the free enzyme (Mannen et al., 2001; Liu et al., 2013; Secundo, 2013). In addition, the immobilization led to an increase in the steric hindrance between the enzyme and the substrate (Ying et al., 2002; Zhang et al., 2012; Rodrigues et al., 2013; Jahangiri et al., 2014; Zhang et al., 2014) which caused a decrease in the activity of the immobilized enzyme. However, the unique structure of the porous microspheres could impart a good microenvironment to the immobilized enzyme molecules, reduce the influence of external adverse factors on the enzyme molecules, and thereby improve the thermal stability (Han et al., 2015; Han et al., 2016).

**FIGURE 3:**
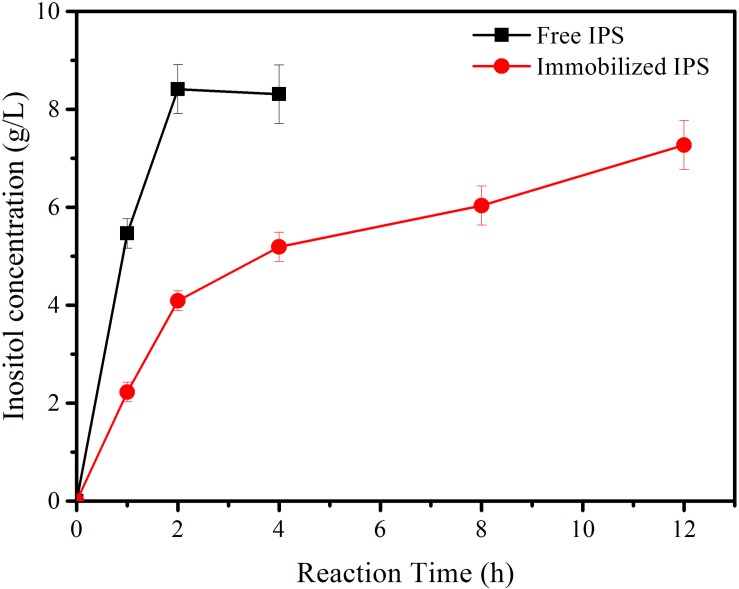
The inositol production as a function of reaction time catalyzed by the reaction systems containing free and immobilized IPS.

**FIGURE 4:**
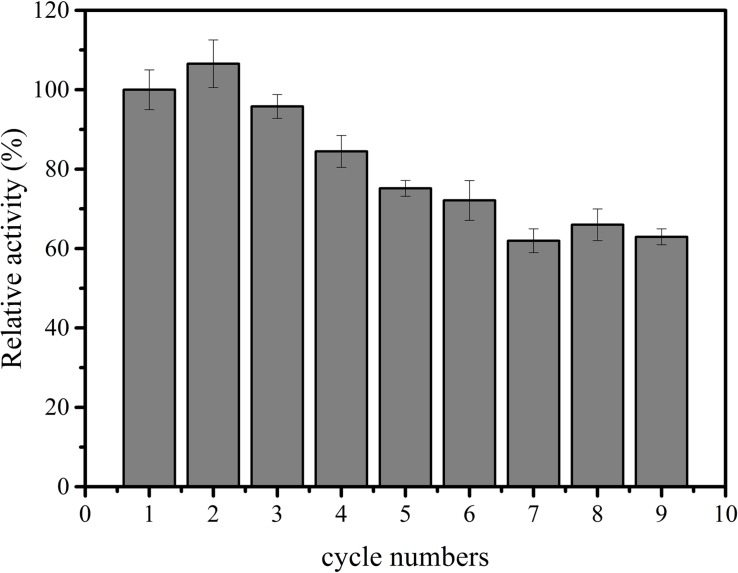
Recycling stability of free and immobilized IPS.

Subsequently, immobilized IPS was used to produce inositol along with the free αGP, PGM, and IMP at 70°C within 12 h. As shown in Figure 3, the product inositol yields of *in vitro* biosystems containing immobilized and free IPS was 70.0 and 81.3%, respectively. These two values are not far away, however, the biosystem containing free enzyme only took 2 h to achieve the yield of 81.3% and the biosystem containing immobilized IPS took 12 h to achieve the yield of 70.0%, this result meant that the reaction rate of the biosystems containing immobilized IPS showed much lower activity than that containing free IPS because of the lower activity of immobilized IPS. Then the recycling stability of immobilized IPS was investigated. As shown in Figure 4, the relative activity for immobilized IPS was approximately 62.9% after 9 cycles, in which cycle period was set to 2 h. The results showed that the immobilized IPS had good recyclability. The results of the catalytic activity and stability of the immobilized IPS described above indicated that the porous microspheres were suitable for immobilization of the rate-limiting enzyme in *in vitro* synthetic enzymatic biosystem for inositol production from maltodextrin.

### Immobilization of αGP, PGM, and IMP

After the immobilization of the rate-limiting enzyme was successfully immobilized on porous microspheres, the other three enzymes were immobilized in the same manner. The enzyme loading amounts of these three enzymes immobilized on porous microspheres were not different from IPS. The enzymatic activities of these three immobilized enzymes were examined. As shown in Table 1, the specific activity values of all the immobilized enzymes showed various degree of decrease compared to their free enzyme counterparts. Specially, the specific activities of immobilized αGP, PGM, and IMP were 47.6, 60.56, 40.8% of their free enzyme counterparts at the assayed conditions (pH 7.2, 70°C), respectively. However, the thermal stabilities of immobilized enzymes was much higher than free enzymes at 70°C or 80°C. As shown in Supplementary Figure S1, the immobilized αGP maintained 89.8% or 85.2% residual activity after incubation for at 70°C 24 h or 80°C for 8 h, respectively, while the free enzyme maintained only 50% residual activity. For PGM, the immobilized enzyme maintained 46.2% or 46.6% residual activity after incubation at 70°C for 18.5 h or at 80°C for 4.5 h, while the free enzyme maintained only 20.9% or 28.7% residual activity, respectively. For IMP, the immobilized enzyme maintained 72.2% or 70.5% residual activity after incubation at 70°C for 24 h or 80°C for 8 h, respectively, while the free enzyme maintained only 59.3% or 37.2% residual activity, respectively. As shown in Table 2, the half-lives of immobilized enzymes for αGP, PGM, and IMP were 2.8, 1.9, and 1.4 times of their free enzyme counterparts at 70°C, while these values were 4.4, 2.5, and 1.8 times at 80°C. These results indicated that all the enzymes in the *in vitro* biosystem for inositol production from starch could be immobilized on porous microspheres.

### Co-immobilization of αGP, PGM, IPS, and IMP for Inositol Production

As mentioned earlier, the co-immobilization of multiple enzymes could reduce the distance of the enzymes, resulting in a high probability of the increased reaction rate. After learning that αGP, PGM, IPS, and IMP could be immobilized by porous microspheres individually, these four enzymes were co-immobilized on porous microspheres for inositol production. All the four enzymes were pre-mixed in the ratio mentioned in Methods section, followed by the immobilization on the porous microspheres. The loading capacity for these four enzymes on porous microspheres was about 155 mg protein/g carrier (aGP 17.6 mg/g carrier; PGM 17.6 mg/g carrier; IPS 105.7 mg/g carrier; and IMP 14.1 mg/g carrier).

**FIGURE 5:**
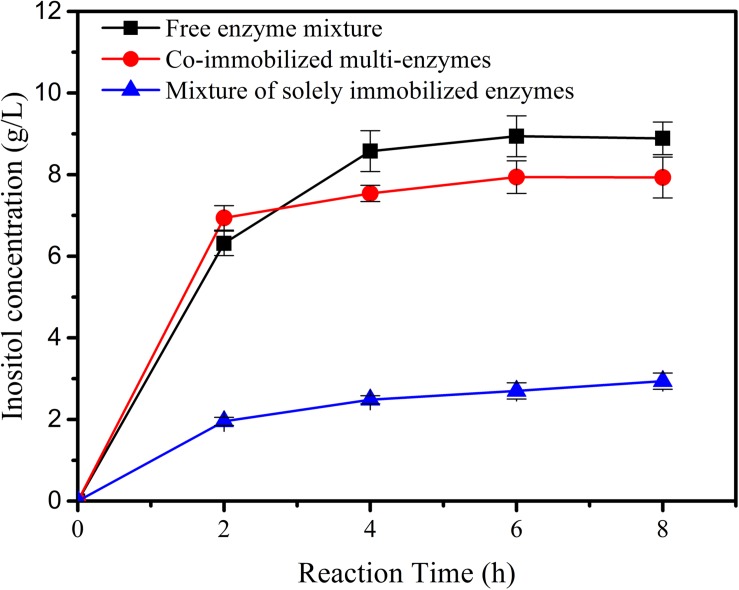
The inositol production as a function of the reaction time catalyzed by free enzyme mixture, co-immobilized multi-enzymes, and mixture of solely immobilized enzymes.

The co-immobilized multi-enzymes on the porous microspheres were used to produce inositol from 1% maltodextrin. The activity of the co-immobilized multi-enzyme system was compared to biosystems containing the mixture of free enzymes or immobilized single enzymes. As shown in Figure 5, inositol yield for co-immobilized multi-enzyme system was 69.4% in the first 2 h, which was slightly higher than that of free enzyme mixture (63.1%). And at hour 8, inositol yield of the immobilized multi-enzyme system was 79.4%, which was a little lower than that of the free enzyme mixture (88.9%). This result indicated that there was no big difference in catalytic efficiency between the co-immobilized enzyme system and the free enzyme system. However, co-immobilized multi-enzymes exhibited a much higher initial reaction rate and product yield than the mixture of the immobilized single enzyme. Generally speaking, immobilized enzymes always showed lower activity than free enzymes, this phenomenon was also appeared in this study. However, if multi-enzymes were immobilized simultaneously, the distance between the cascade enzymes became short, this proximity effect might result in the fast utilization of intermediates (You et al., 2012; You and Zhang, 2013; Bao et al., 2018), and the well-developed pore structure in the microspheres provided excellent microenvironment for the transfer of substrate, intermediates and product, resulting in the high reaction rate of co-immobilized multi-enzyme system.

**FIGURE 6:**
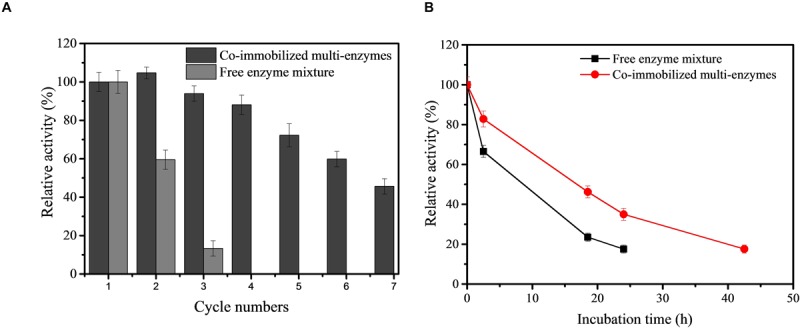
Recycling stability of free enzyme mixture and co-immobilized multi-enzymes **(A)** and the thermostability of free enzyme mixture and co-immobilized multi-enzymes **(B)**.

Given the potential industrial application of our *in vitro* synthetic enzymatic biosystem for inositol production, therefore, the recovery stability of the co-immobilized multi-enzyme system was investigated. After the enzymes were immobilized, the co-immobilized enzymes could be separated from the reaction solution by simple centrifugation after the production process. On the basis of the result of Figure 5, the reaction time of each cycle was set at 2 h. As indicated in Figure 6A, the co-immobilized multi-enzymes system retained about 45.6% of its initial activity after 7th cycles of enzyme use. However, free enzyme mixture was recycled through the ultrafiltration and only maintained a 13.3% relative yield of inositol production after 3 cycles. The recycling of multiple enzymes help to reduce the production costs of *in vitro* synthetic enzymatic biosystems. In addition, the immobilization of multiple enzymes could help simplify the separation process and improve product quality during product separation (Sheldon and Brady, 2018). The cost of free enzyme mixture was $150/kg based on the high density fermentation in T&J-A type 5L^∗^2 Parallel Bioreactor in our lab. The cost of co-immobilized multi-enzymes was $750/kg based on the materials and the operation process. Based on the experimental data in this study, for free enzyme mixture, 0.077 *g* of enzymes was needed to produce one gram inositol, so the cost of 1 kg of inositol was about $11.55. For co-immobilized multi-enzymes, 0.013 *g* of enzymes was needed to produce one gram inositol, so the cost of 1 kg of inositol was about $9.75. Compared to free enzyme mixture, the cost of co-immobilized multi-enzymes was reduced by 15.6% to produce equal amount of inositol. The co-immobilization of multiple enzymes not only increased the recycling of multiple enzymes, decreasing the enzyme cost for biomanufacturing, and but also improved the thermal stability of multiple enzymes. The thermal stability of the co-immobilized multi-enzymes at 70°C was measured. As shown in Figure 6B, after incubation at 70°C for 24 h, the inositol concentration produced by the treated free multi-enzymes was only 16.7% of that produced by the untreated free multi-enzymes. However, after incubation at 70°C for 24 h, the inositol concentration produced by the treated co-immobilized multi-enzymes was 34.9% of that produced by the untreated co-immobilized multi-enzymes. Compared with the half-life of free multi-enzymes being only 9.7 h, the half-life of co-immobilized multi-enzymes could be extended to 17.3 h. In addition, no protein was detected in the supernatant of co-immobilized multi-enzymes after 24 h of incubation. Therefore, the decrease in the remaining enzyme activity in recycling experiments was due to heat inactivation. Moreover, the pH stabilities of free enzyme mixture and co-immobilized multi-enzymes were investigated. Both free enzyme mixture and co-immobilized multi-enzymes maintained high catalytic activity under alkaline conditions, while the catalytic activity decreased significantly under acidic conditions. Compared with free enzyme mixture, the pH and temperature stability of co-immobilized multi-enzymes were both improved (Supplementary Figure S2).

## Conclusion

In conclusion, porous microspheres were used as carriers for immobilizing multiple enzymes to produce inositol from starch. Compared to free enzyme mixture, co-immobilized multi-enzymes on porous microspheres showed higher thermal stability and recycling stability. Despite the activity loss of every individual enzyme on porous microspheres, co-immobilized multi-enzyme biosystem showed comparable reaction rate with the free enzyme mixture, which may due to the effect of substrate channeling. Because enzyme cost can be decreased by enzyme co-immobilization, this study shed light on reducing the product cost of *in vitro* synthetic enzymatic biosystems in industrial scale by enzyme co-immobilization.

## Data Availability Statement

All datasets generated for this study are included in the article/Supplementary Material.

## Author Contributions

PH and CY designed the experiments, analyzed the data, and wrote the manuscript. PH and XZ performed the experiments.

## Conflict of Interest

The authors declare that the research was conducted in the absence of any commercial or financial relationships that could be construed as a potential conflict of interest.
