# Supplementary material for: Efficient Multi-Enzymes Immobilized on Porous Microspheres for Producing Inositol From Starch
Source: Front Bioeng Biotechnol. 2020 May 5;8:380. doi: 10.3389/fbioe.2020.00380 (PMC7232586; doi:10.3389/fbioe.2020.00380)
Supplement: Supplementary file 1 [file Table_1.DOCX]

**Efficient Multi-enzymes immobilized on porous microspheres for producing inositol from starch**

Pingping Han, Xigui Zhou, Chun You

Tianjin Institute of Industrial Biotechnology, Chinese Academy of Science, 32 West 7^th^ Avenue, Tianjin Airport Economic Area, Tianjin 300308, China;

Telephone: +86 022-2482-8789; fax: 001-540-231-3199; e-mail: you_c@tib.cas.cn

**Acknowledgements**

The authors thank the financial support from the National Natural Science Foundation of China (Grant No. 21778073)

a)

b)

c)

Figure S1. Thermostability of free and immobilized enzymes (70 ^o^C or 80 ^o^C). a) aGP; b) PGM; c) IMP.


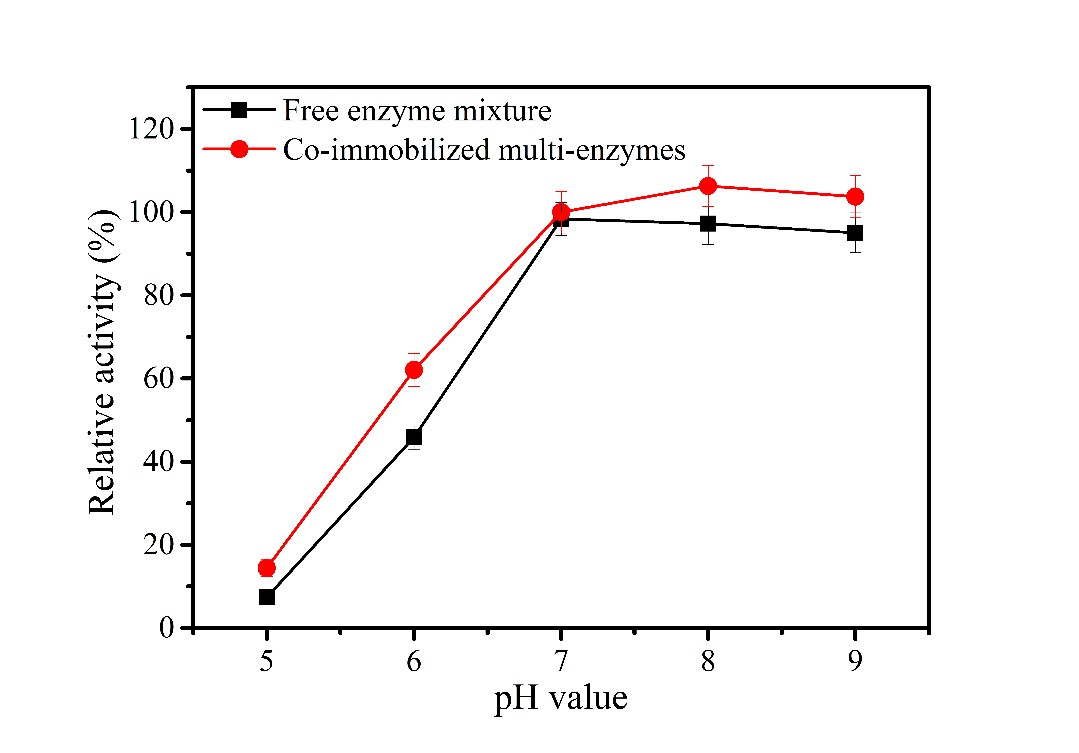


Figure S2. pH stability of free enzyme mixture and co-immobilized multi-enzymes.

Both free enzyme mixture and co-immobilized multi-enzymes maintained high catalytic activity under alkaline conditions, while the catalytic activity decreased significantly under acidic conditions. Compared with free enzyme mixture, the pH stability of co-immobilized multi-enzymes was improved. For example, the co-immobilized multi-enzymes maintained a relative enzyme activity of 14.4%, while free enzyme mixture only had a relative enzyme activity of 7.3% at pH 5. The co-immobilized multi-enzymes maintained 102.4% relative enzyme activity, while free enzyme mixture maintained 95.4% relative enzyme activity at pH 9.
